# Metabolic health during a randomized controlled lifestyle intervention in women with PCOS

**DOI:** 10.1530/EJE-21-0669

**Published:** 2021-10-28

**Authors:** Alexandra Dietz de Loos, Geranne Jiskoot, Annemerle Beerthuizen, Jan Busschbach, Joop Laven

**Affiliations:** 1 Division of Reproductive Endocrinology and Infertility, Department of Obstetrics and Gynaecology, Erasmus MC, Rotterdam, the Netherlands; 2 Department of Psychiatry, Section Medical Psychology and Psychotherapy, Erasmus MC, Rotterdam, the Netherlands

## Abstract

**Context:**

Women with polycystic ovary syndrome (PCOS) have an increased risk of metabolic syndrome (MetS). Both PCOS and MetS are associated with excess weight.

**Objective:**

To examine the effect of a three-component lifestyle intervention (LSI) with or without short message service (SMS+ or SMS−, respectively) on the prevalence and severity of MetS and metabolic parameters, compared to care as usual (CAU).

**Design:**

Randomized controlled trial.

**Methods:**

Women diagnosed with PCOS and a BMI >25 kg/m^2^ (*n* = 183) were either assigned to a 1-year three-component (cognitive behavioural therapy, diet, and exercise) LSI, with or without SMS support, or to CAU which provided weight-loss advice only. Main outcome measures included changes in the prevalence of MetS, the continuous MetS severity z-score (cMetS z-score), metabolic parameters, and the impact of weight loss.

**Results:**

After 1 year, the decrease in the cMetS z-score was greater in the SMS+ group than the CAU group (−0.39, *P*  = 0.015). The prevalence of MetS changed with −21.6% (*P*  = 0.037), −16.5% (*P*  = 0.190), and +7.0% (*P*  = 0.509) in both LSI groups and CAU group, respectively. A *post hoc* analysis for both LSI groups combined vs CAU resulted in a MetS difference of −25.9% (*P*  = 0.046). Moreover, weight loss *per se* resulted in significantly favourable effects on all metabolic parameters.

**Conclusions:**

This three-component LSI was more successful in improving metabolic health compared to CAU. Therefore, we recommend this intervention to women with PCOS and excess weight, provided that a clinically relevant weight loss is being pursued.

## Introduction

With a prevalence of 8–13% (1, 2, 3, 4, 5), polycystic ovary syndrome (PCOS) is the most common endocrine disorder in women during their reproductive lifespan. Women with PCOS present more often with individual metabolic features such as elevated blood pressure (BP), enlarged waist circumference (WC), and an impaired glucose tolerance (6). Other key features of metabolic syndrome (MetS) are elevated triglyceride (TG) levels and reduced high-density lipoprotein (HDL).

The increased prevalence of all these individual metabolic features leads to a higher prevalence of MetS among women with PCOS. For example, women with PCOS are especially prone to central obesity (7). Moreover, obesity exacerbates many of the metabolic abnormalities that are already associated with PCOS, such as insulin resistance and lipid abnormalities (1, 8, 9). It has been found that insulin levels and lipid profiles were most severely affected in the subgroup of PCOS cases that had both hyperandrogenism and a BMI of ≥25 kg/m^2^ (10). Overall, women with PCOS have more than a three-fold increase in MetS prevalence compared to women without PCOS (9, 11).

MetS is considered to be a pathological state associated with cardiovascular disease (12). Therefore, it is desirable to detect and treat MetS before irreversible cardiovascular events and/or diabetes mellitus will occur. Some believe that several cardiovascular risk factors associated with PCOS, such as obesity, diabetes mellitus, hypertension, and dyslipidemia, are driven by insulin resistance as the same pathogenic mechanism (13). Insulin resistance and hyperandrogenism are believed to affect the lipid profile among women with PCOS, causing dyslipidemia even in non-obese women with PCOS (14). A therapeutic approach could be insulin-sensitizing agents such as inositols or metformin (13, 15), which seem to have a beneficial effect on metabolic derangements associated with PCOS. Also, others believe that there is a therapeutic role of foods and nutrients of specific dietary patterns (e.g. the Mediterranean diet) which have positive effects on the clinical severity of PCOS, improving inflammatory status, insulin resistance, and hyperandrogenemia (16). Physical activity is also believed to improve insulin resistance, cardiovascular, and metabolic diseases (17, 18). In general, we can conclude that lifestyle adjustments are necessary to improve the metabolic status of women with PCOS.

All five components of MetS are alleviated in the general population by even modest amounts of weight loss achieved with diet and exercise (19). Weight management by a three-component (diet, exercise, and behavioural therapy) lifestyle intervention (LSI) is currently the first-line treatment for women with PCOS (1), despite the well-known difficulties with adherence in LSI. Dropout is a common phenomenon, and discontinuation rates varying between 12 and 47% in LSI studies in overweight and obese women with PCOS have been reported (20). Nevertheless, previous short-term one or two-component LSIs in women with PCOS have described improvements in metabolic features such as WC, total cholesterol, LDL cholesterol, and fasting insulin (21). However, evidence is still lacking on changes in the prevalence and severity of MetS in overweight and obese women with PCOS as a result of long-term three-component LSIs.

We performed a randomized controlled trial (RCT) in overweight and obese women with PCOS that compared the effects of a 1-year three-component LSI with or without short message service (SMS) support to the effects of care as usual (CAU) (22). The aim of this study was to evaluate the effects of the intervention on the prevalence of MetS and its diagnostic components, as well as on the severity of MetS over the course of the study.

## Subjects and methods

### Trial design

Between August 2, 2010, and March 11, 2016, we conducted a randomized controlled 1-year three-component LSI, which was approved by the Medical Research Ethics Committee of the Erasmus MC in Rotterdam (MEC2008-337) and registered by clinical trial number: NTR2450 (www.trialregister.nl). The trial consisted of three arms: (i) 1-year three-component LSI with SMS (SMS+), (ii) 1-year three-component LSI without SMS (SMS−), and (iii) CAU. The protocol was published previously (22).

In this study, we examined the effect of the LSI groups SMS+ and SMS− compared to CAU on the MetS and on the different metabolic parameters (homeostasis model assessment (HOMA-IR), BP, WC, fasting glucose, fasting insulin, lipids). Additionally, the effect of SMS support within the LSI was analysed, as well as the effect of the RCT on the continuous MetS severity z-score. Finally, different *post hoc* analyses were performed including an evaluation of the longitudinal effect after pooling both LSI groups (SMS+ and SMS−) compared to CAU on the above-mentioned outcome measures and mediation analyses. We also investigated the effect of weight change *per se* (all groups combined) on the MetS and metabolic parameters.

Outcome measures were assessed every 3 months starting at baseline until the endpoint at 12 months.

### Participants

Women were enrolled at the outpatient clinic within the division of Reproductive Endocrinology and Infertility of the Department of Obstetrics and Gynaecology, at the Erasmus MC, the Netherlands. Inclusion criteria comprised women who were actively trying to conceive with a BMI > 25 kg/m^2^, between 18 and 38 years of age, and a diagnosis of PCOS according to the Rotterdam 2003 consensus criteria (23). Exclusion criteria comprised the lack of proficient use of the Dutch language, severe mental illness, adrenal diseases or ovarian tumours, and other causes leading to androgen excess, and other malformations of the internal genitalia. With the use of an extensive endocrine screening, which is specified below, women with other secondary endocrine (e.g. hypothyroidism, Cushing’s disease, hypothalamic obesity, hypogonadism, and insulinoma), drug-related, and, when indicated, genetic causes of secondary obesity were identified and excluded. Participants discontinued the study if pregnancy was achieved.

After they provided written informed consent, women were randomly assigned in a 1:1:1 ratio to one of the three arms of the study: SMS+, SMS−, or CAU. Randomization was performed using a computer-generated random numbers table. The sample size was calculated based on an expected dropout proportion of 40%, resulting in a minimum of 60 participants in each group.

PCOS was diagnosed when at least two of the following key features were present: ovulatory dysfunction (cycle interval length > 35 or < 21 days), clinical (modified Ferriman Gallwey score ≥ 5), and/or biochemical (testosterone measured with RIA: free androgen index (FAI) cut off > 4.5 and/or total testosterone > 3.0, testosterone measured with liquid chromatography-tandem mass spectrometry (LC-MS/MS): FAI cut off > 2.9 and/or total testosterone > 2.0 nmol/L) hyperandrogenism and polycystic ovarian morphology (PCOM; ≥12 follicles (measuring 2–9 mm in diameter) and/or ovarian volume > 10 cm^3^ in at least one ovary using an ultrasound machine with a transvaginal probe of less than 8 MHz) (1, 23). MetS was defined according to the National Cholesterol Education Programme (NCEP) definition when at least three of the following features were present: WC ≥ 88 cm, fasting glucose ≥ 6.1 mmol/L, BP > 129/84 mmHg, HDL < 1.3 mmol/L, and TG ≥ 1.7 mmol/L (11).

### Clinical and endocrine assessments

All three groups underwent five extensive standardized endocrine assessments after an overnight fast, which included general medical, obstetric and family history, and anthropometric measurements. Height was measured using a wall-mounted stadiometer (Seca 220; Seca, Hamburg, Germany), body weight was measured using a calibrated scale (Seca 877; Seca, Hamburg, Germany), and BMI (kg/m^2^) was calculated. WC was measured in standing position, without heavy outer garments, midway between the lower rib and iliac crest according to the NCEP guidelines (11). BP was measured, and a transvaginal ultrasound was performed (probe of less than 8 MHz). Fasting blood samples were collected and assessed on insulin, glucose, gonadotropins, sex steroids, thyroid hormones, lipid profile, and adrenal steroids. Insulin was measured using the Roche Modular E170 assay (Roche Diagnostics) with intra-assay and inter-assay coefficients of variation (CV) of <2 and <4%, respectively. The COBAS 8000 Modular Analyser (Roche Diagnostics GmbH) was used to measure glucose (intra-assay CV < 0.8% and inter-assay CV < 1.4%) and cholesterol, HDL, LDL, and TG (intra-assay CV < 1.1% and inter-assay CV < 2.1%). The HOMA-IR was used to assess insulin resistance. Insulin was converted from pmol/L to mU/L. Subsequently, the HOMA-IR was calculated as: fasting insulin (mU/L) × fasting glucose (mmol/L) / 22.5 (24). Insulin resistance was present when 1/HOMA-IR < 0.47 (24).

### Continuous metabolic syndrome severity z-score

The continuous MetS severity z-score (cMetS z-score) was calculated to provide a clinically accessible and interpretable continuous measurement in order to identify participants who are at higher risk for MetS-related diseases as well as to follow changes within individuals over time. The MetS z-score was derived from a confirmatory factor analysis which examined the weighted contribution of each of the five components of the MetS. This analysis also allowed for the correlations between MetS components to be different by sex and ethnicity. Eventually, this resulted in different equations for sex- and ethnic-specific MetS risk z-score with mean 0 and s.d. equals 1. Scores above 0 are associated with an increased risk for future cardiometabolic disease (25, 26, 27, 28).

### Three-component lifestyle intervention and control group (CAU)

The LSI lasted 12 months and covered three components in twenty 2.5-h group meetings: cognitive behavioural therapy (CBT), diet, and exercise. CBT techniques were used to, for example, create awareness and to restructure dysfunctional thoughts about lifestyle, weight (loss), and self-esteem. The ‘Dutch Food Guide’ was used as a guideline for healthy eating (29), and the global recommendations for physical activity by the World Health Organization (30) formed the basis for the exercise component of the LSI. After 3 months, half of the LSI group received additional patient-tailored SMS feedback. Participants sent weekly self-monitored information regarding their diet, physical activity, and emotions by SMS. A semi-automated software programme generated feedback in response to the incoming messages with the goal to encourage positive behaviour and provide social support. Participants also received two additional messages per week addressing eating behaviour and physical activity. Examples of types of messages are further specified in the study protocol (22). In order to get acquainted with, and to evaluate the acceptability of the LSI, we tested the LSI in a pilot group (*n* = 26) before enrolling participants for the study. These data were not used for the current study.

CAU(control) comprised an advice to adopt a healthy lifestyle and to lose weight by methods of their own choosing (e.g. visit a dietician or gym) and consultations with their treating physician.

### Statistical methods

Between-group (SMS+ vs CAU, SMS− vs CAU, and SMS+ vs SMS−) and within-group analyses on the prevalence of MetS, metabolic parameters, and cMetS z-score were performed with multilevel logistic and linear regression models based on the intention-to-treat principle. Mixed modelling can efficiently deal with missing data and unbalanced time points (31). These analyses included an upper level and a lower level, which represented the participants and their repeated measures, respectively. Study group, logarithmic time, and interactions were included as independent variables, and a bootstrap procedure with 10 000 samples was performed in case of a non-normal distribution.

#### Post hoc analyses

Mixed modelling was also applied for the *post hoc* analyses. First, the SMS+ and SMS− groups were pooled in order to evaluate the complete LSI group vs CAU. We also pooled all three groups to analyse all cases who changed in body weight (with % of body weight as a continuous variable) and their effect on the outcome measures (MetS, cMetS z-score, and different metabolic parameters).

With mediation analyses, we evaluated the relationship between the independent variable (LSI) and dependent variable (MetS) with potential mediators (weight, androgens, sex hormone-binding globulin (SHBG), insulin, and HOMA-IR). Pathways α, β, τ, and τ’ were analysed with mixed modelling, and a mediation ratio with coinciding *P*-value was calculated with the following equations:


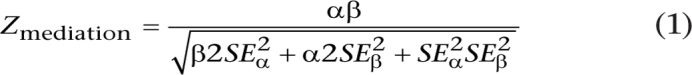



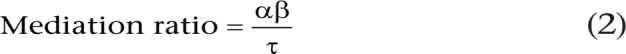


IBM SPSS statistics version 25.0 was used for multilevel linear analyses including bootstrap procedure. SAS version 9.4 (SAS Institute Inc., Cary, NC, USA) was used for multilevel logistic regression analyses. A *P*-value of <0.05 was considered statistically significant.

## Results

**Figure 1:**
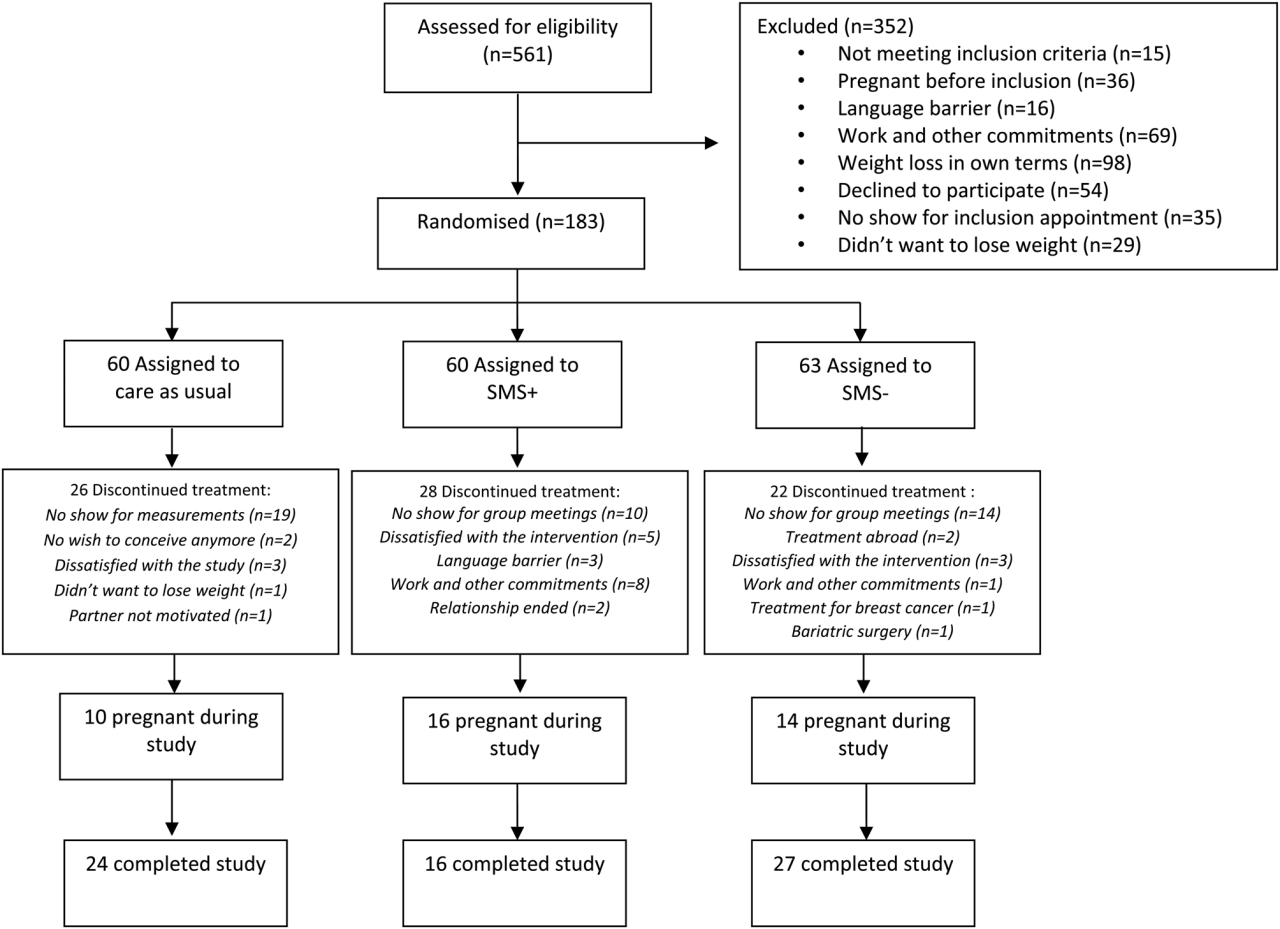
CONSORT flowchart.

Between August 2, 2010, and March 11, 2016, 561 eligible women were identified to participate in the trial, of whom 26 women were included in a pilot study and 352 women were excluded with reasons further specified in Fig. 1. This resulted in 183 women who were randomly assigned to the SMS+ group (*n* = 60), SMS− group (*n* = 63), and CAU group (*n* = 60). Eventually, 27, 16, and 24 women completed the study for the SMS−, SMS+, and CAU groups respectively, overall resulting in a 36.6% completion rate. A total of 485 measurements were available for these intention-to-treat analyses. Baseline characteristics are presented in Table 1. MetS was present in 41.4% in the SMS+ group, in 48.3% in the SMS− group, and in 38.5% in the CAU group. Overall, median age was 29 years (IQR 26–32), and median BMI was 32.8 kg/m^2^ (IQR 30.1–36.1). Mean weight loss after 1 year was 7.87 kg in SMS+, 4.65 kg in SMS−, and 2.32 kg in CAU (within all groups, *P* < 0.001). The proportion of women who achieved >5% and >10% weight reduction was 85.7 and 45.9% within SMS+, 52.8 and 12.2% within SMS−, and 21.8 and 6.8% within CAU, respectively (32).

**Table 1:** Baseline characteristics. Values are displayed as *n* (%) or as medians (interquartile range).

	Lifestyle intervention		Care as usual (*n* = 60)
	SMS+ (*n* = 60)	SMS− (*n* = 63)	
Metabolic syndrome	24 (41.4%)	29 (48.3%)	20 (38.5%)
Insulin resistance	39 (66.1%)	40 (65.6%)	40 (67.8%)
Nulliparous	47 (79.7%)	47 (75.8%)	44 (75.9%)
Caucasian	30 (50.0%)	21 (35.0%)	25 (42.4%)
Smoking	13 (21.7%)	11 (17.7%)	14 (23.7%)
Alcohol consumption	12 (20.0%)	15 (24.2%)	19 (32.2%)
Education			
Low	5 (8.3%)	5 (8.2%)	8 (14.3%)
Intermediate	33 (55.0%)	34 (55.7%)	35 (62.5%)
High	22 (36.7%)	22 (36.1%)	13 (23.2%)
cMetS z-score	0.36 (0.02 to 0.85)	0.37 (−0.01 to 0.82)	0.31 (−0.11 to 0.66)
Waist (cm)	101 (93 to 107)	96 (89 to 109)	96 (89 to 109)
SBP (mmHg)	120 (112 to 125)	121 (115 to 130)	120 (110 to 125)
DBP (mmHg)	80 (74 to 81)	80 (75 to 84)	79 (70 to 84)
Glucose (mmol/L)	5.0 (4.7 to 5.3)	5.2 (4.8 to 5.4)	5.0 (4.7 to 5.3)
Insulin (pmol/L)	87 (51 to 122)	103 (54 to 148)	89 (62 to 123)
HOMA-IR	2.79 (1.73 to 4.27)	3.28 (1.75 to 5.21)	2.84 (1.99 to 4.07)
HDL (mmol/L)	0.93 (0.79 to 1.05)	0.90 (0.76 to 1.10)	0.85 (0.73 to 0.98)
LDL (mmol/L)	3.17 (2.67 to 3.83)	3.16 (2.65 to 3.85)	3.17 (2.61 to 3.73)
Cholesterol (mmol/L)	4.8 (4.2 to 5.4)	4.7 (4.2 to 5.4)	4.8 (4.0 to 5.2)
Triglycerides (mmol/L)	1.12 (0.83 to 1.69)	1.23 (0.91 to 1.70)	1.27 (0.83 to 1.78)
Age (years)	28 (26 to 32)	30 (27 to 33)	28 (26 to 32)
Weight (kg)	95 (85 to 106)	89 (80 to 104)	84 (79 to 97)
BMI (kg/m^2^)	33.5 (30.9 to 37.1)	33.6 (30.4 to 36.0)	30.6 (29.3 to 34.3)
Age of menarche (years)	12 (12 to 14)	12 (11 to 13)	12 (11 to 13)

cMetS z-score, continuous metabolic syndrome z-score; DBP, diastolic blood pressure; HDL, high-density lipoprotein; HOMA-IR, homeostatic model assessment for insulin resistance; IQR, interquartile range; LDL, low-density lipoprotein; SBP, systolic blood pressure; SMS−, lifestyle intervention without SMS support; SMS+, lifestyle intervention with SMS support.

### Between-group estimates after 12 months (SMS+ vs CAU, SMS− vs CAU, and SMS+ vs SMS−)

The MetS risk z-score decreased from 0.44 to 0.02 in SMS+, from 0.41 to 0.20 in SMS−, and from 0.39 to 0.36 in CAU. The difference between SMS+ and CAU after 12 months was significant (−0.39, *P*  = 0.015). The SBP decreased from 120 to 115 mmHg in the SMS+ group, from 121 to 116 mmHg in the SMS− group, and increased from 119 to 121 mmHg in the CAU group. The difference at 12 months was significant in favour of both the SMS+ (−7 mmHg, *P*  = 0.039) and SMS− (−6 mmHg, *P*  = 0.013) group when compared to CAU. Subsequently, the prevalence of having a BP ≥ 129/84 mmHg as a diagnostic criterion for MetS decreased from 38.1% at baseline to 15.7% at 12 months in the SMS− group and increased from 29.0 to 36.2% in the CAU group, resulting in a significant difference of 29.5% (*P*  = 0.020) between the groups at 12 months. However, a difference in mean HDL serum levels of 0.10 mmol/L in favour of the CAU group (from 0.87 mmol/L at baseline to 0.93 mmol/L at 12 months) compared to the SMS+ group (from 0.94 mmol/L at baseline to 0.90 mmol/L at 12 months) was also observed (*P*  = 0.018), see Table 2.

**Table 2:** Difference in the metabolic syndrome and metabolic parameters between study groups at 12 months. Differences were tested with multilevel logistic regression analyses for categorical variables and with multilevel linear regression analyses for continuous variables, combined with a bootstrap procedure in case of a non-normal distribution.

	SMS+ vs CAU difference		SMS− vs CAU difference		SMS+ vs SMS− difference	
	Value	*P* value	Value	*P* value	Value	*P* value
Metabolic syndrome (%)	−23.7	0.146	−28.6	0.052	5.1	0.808
Waist ≥ 88 cm (%)	−0.7	0.763	−2.8	0.505	1.5	0.810
Glucose ≥ 6.1 mmol/L (%)	−5.6	0.377	−6.5	0.254	0.1	0.993
BP ≥ 129/84 mmHg (%)	−17.2	0.233	−29.5	**0.020**	12.4	0.408
HDL <1.3 mmol/L (%)	5.1	0.224	5.4	0.415	2.4	0.429
TG ≥ 1.7 mmol/L (%)	−1.6	0.910	−7.1	0.532	5.2	0.686
Insulin resistance (%)	−10.3	0.492	−1.8	0.890	−7.2	0.642
Metabolic parameters						
cMetS z-score	−0.39	**0.015**	−0.18	0.172	−0.17	0.159
HOMA-IR	−0.28	0.683	−0.05	0.942	0.29	0.591
SBP (mmHg)	−7	**0.039**	−6	**0.013**	0	0.879
DBP (mmHg)	−4	0.084	−3	0.109	0	0.867
Waist (cm)	−3.2	0.400	1.3	0.648	4.40	0.201
Glucose (mmol/L)	−0.3	0.153	−0.2	0.206	0.03	0.862
Insulin (pmol/L)	−3	0.896	6	0.781	10.46	0.474
Cholesterol (mmol/L)	−0.4	0.054	−0.2	0.265	0.2	0.348
HDL (mmol/L)	−0.10	**0.018**	−0.04	0.322	0.06	0.218
LDL (mmol/L)	−0.18	0.284	−0.06	0.700	0.12	0.485
TG (mmol/L)	−0.17	0.333	−0.22	0.139	−0.01	0.946

BP, blood pressure; CAU, care as usual; cMetS z-score, continuous metabolic syndrome z-score; DBP, diastolic blood pressure; HDL, high-density lipoprotein; HOMA-IR, homeostatic model assessment for insulin resistance; LDL, low-density lipoprotein; SBP, systolic blood pressure; SMS−, lifestyle intervention without SMS support; SMS+, lifestyle intervention with SMS support; TG, triglycerides. Statistically significant values (*P<*0.05) are presented in bold.

### Within-group estimates after 12 months

Table 3 provides all within-group changes from baseline to 12 months. The prevalence of MetS decreased within SMS− from 49.8 to 28.2% (−21.6%, *P*  = 0.037) after 12 months. Within the SMS+ group, the prevalence of MetS decreased from 41.9 to 25.3% (−16.5%, *P*  = 0.190) and increased in CAU from 37.9 to 44.8% (+7.0%, *P*  = 0.509). The cMetS severity z-score decreased within the SMS+ (−0.42, *P*  = 0.002) and SMS− group (−0.21, *P*  = 0.027) but not significant within CAU (−0.03, *P*  = 0.733).

**Table 3:** Within-group changes in metabolic syndrome and metabolic parameters from baseline to 12 months. Differences were tested with multilevel logistic regression analyses for categorical variables and with multilevel linear regression analyses for continuous variables, combined with a bootstrap procedure in case of a non-normal distribution.

Group	At baseline	At 3 months	At 6 months	At 9 months	At 12 months	Change	*P* value within
Metabolic syndrome (%)							
SMS+	41.9	32.4	28.9	26.8	25.3	−16.5	0.190
SMS−	49.8	37.6	32.9	30.2	28.2	−21.6	**0.037**
CAU	37.9	41.6	43.1	44.1	44.8	7.0	0.509
Metabolic parameters							
cMetS z-score							
SMS+	0.44	0.20	0.11	0.06	0.02	−0.42	**0.002**
SMS−	0.41	0.19	0.16	0.17	0.20	−0.21	**0.027**
CAU	0.39	0.22	0.23	0.28	0.36	−0.03	0.733
HOMA-IR							
SMS+	3.33	3.12	3.04	2.99	2.95	−0.38	0.329
SMS−	3.79	3.70	3.67	3.65	3.63	−0.15	0.658
CAU	3.87	3.81	3.79	3.78	3.76	−0.11	0.842
SBP (mmHg)							
SMS+	120	117	116	115	115	−5	0.053
SMS−	121	119	117	117	116	−5	**0.010**
CAU	119	120	120	120	121	1	0.450
DBP (mmHg)							
SMS+	78	76	75	75	74	−4	**0.034**
SMS−	79	77	77	76	76	−3	**0.040**
CAU	78	78	78	78	78	0	0.821
Waist (cm)							
SMS+	102.9	98.4	96.5	95.4	94.5	−8.4	**0.013**
SMS−	100.1	98.1	97.2	96.7	96.3	−3.7	0.097
CAU	100.3	97.6	96.5	95.8	95.2	−5.1	**0.016**
Glucose (mmol/L)							
SMS+	5.1	5.0	5.0	5.0	5.0	−0.2	0.150
SMS−	5.2	5.1	5.1	5.1	5.1	−0.1	0.371
CAU	5.0	5.1	5.1	5.1	5.2	0.1	0.375
Insulin (pmol/L)							
SMS+	100	94	92	90	89	−11	0.376
SMS−	111	110	110	110	110	−2	0.842
CAU	118	114	113	112	111	−8	0.700
Cholesterol (mmol/L)							
SMS+	4.7	4.5	4.4	4.4	4.3	−0.4	**0.009**
SMS−	4.8	4.7	4.7	4.6	4.6	−0.2	0.092
CAU	4.7	4.7	4.7	4.7	4.7	0.0	0.836
HDL (mmol/L)							
SMS+	0.94	0.92	0.91	0.90	0.90	−0.04	0.200
SMS−	0.95	0.96	0.96	0.96	0.97	0.02	0.643
CAU	0.87	0.90	0.92	0.92	0.93	0.06	**0.022**
LDL (mmol/L)							
SMS+	3.21	3.05	2.99	2.94	2.91	−0.29	**0.027**
SMS−	3.24	3.15	3.11	3.09	3.07	−0.17	0.115
CAU	3.25	3.19	3.16	3.15	3.14	−0.11	0.281
TG (mmol/L)							
SMS+	1.33	1.27	1.25	1.23	1.22	−0.11	0.441
SMS−	1.39	1.31	1.28	1.26	1.24	−0.15	0.065
CAU	1.39	1.42	1.43	1.44	1.45	0.06	0.593

CAU, care as usual; cMetS z-score, continuous metabolic syndrome Z score; DBP, diastolic blood pressure; HDL, high-density lipoprotein; HOMA-IR, homeostatic model assessment for insulin resistance; LDL, low-density lipoprotein; SBP, systolic blood pressure; SMS−, lifestyle intervention without SMS support; SMS+, lifestyle intervention with SMS support; TG, triglyceride. Statistically significant values (*P<*0.05) are presented in bold.

Further improvements in the LSI groups included a decrease in SBP in the SMS− group (−5 mmHg, *P*  = 0.010), a decrease in DBP within the SMS+ (−4 mmHg, *P*  = 0.034) and SMS− (−3 mmHg, *P*  = 0.040) groups, a decrease in WC within the SMS+ group (−8.4 cm, *P*  = 0.013), and a decrease in cholesterol (−0.4 mmol/L, *P*  = 0.009) but also in LDL (−0.29 mmol/L, *P*  = 0.027) within the SMS+ group. The CAU group only significantly improved in WC (−5.1 cm, *P*  = 0.016) and HDL (+0.06 mmol/L, *P*  = 0.022) after 12 months.

### *Post hoc* analysis; between-group estimates after 12 months (LSI vs CAU)

**Figure 2:**
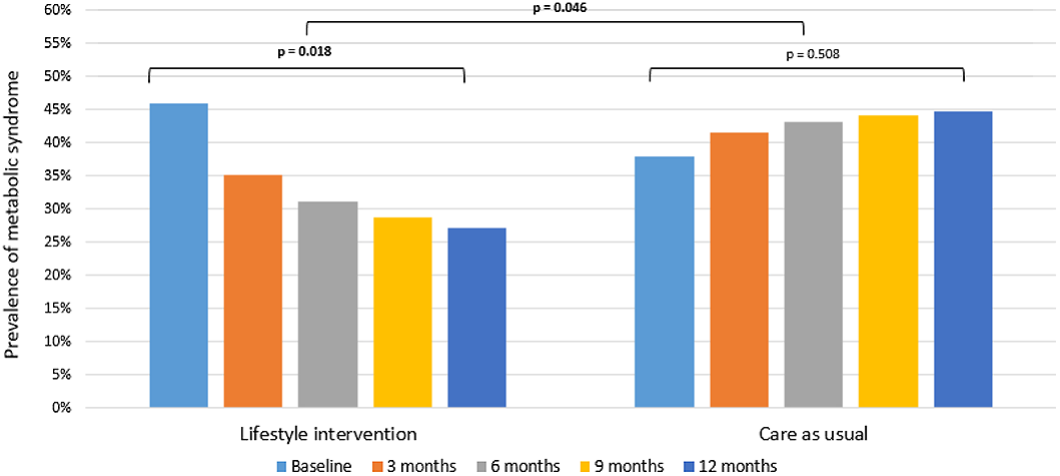
Changes in the prevalence of the metabolic syndrome over time for the lifestyle intervention groups combined compared to the care as usual group. Note: *Post hoc* analysis. Differences were tested with multilevel logistic regression.

**Figure 3:**
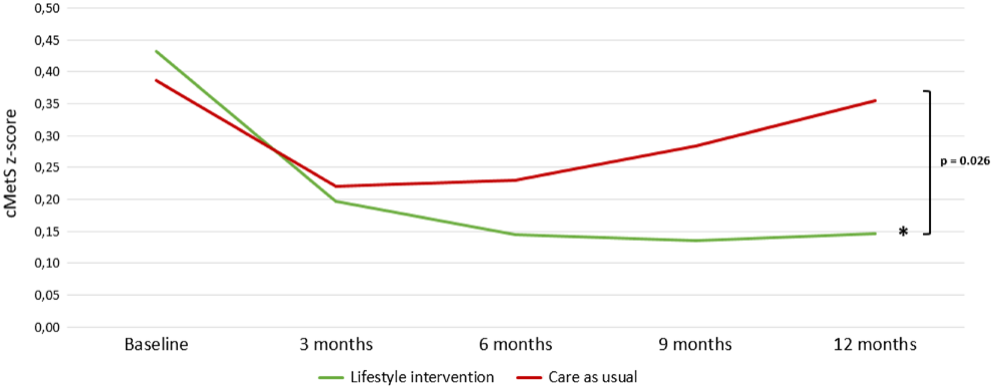
Changes in the continuous metabolic syndrome z-score over time for the lifestyle intervention groups combined compared to the care as usual group. Note: *Post hoc* analysis. Differences were tested with multilevel linear regression. *Indicates statistical significance (*P* < 0.001) for within-group changes. cMetS z-score, continuous metabolic syndrome z-score.

As we observed promising results in both the SMS+ and SMS− groups, we have combined the groups for a *post hoc* analysis in order to evaluate the effect of the LSI vs CAU. Indeed, the prevalence of MetS was statistically significant and more reduced with −25.9% (*P*  = 0.046) within the LSI (from 46.0% at baseline to 27.1% at 12 months, −18.9%) compared to an increase in the CAU group (from 37.9% at baseline to 44.8% at 12 months, +7.0%), see Fig. 2. Also, the cMetS risk z-score reduced −0.25 more (*P*  = 0.030) within the LSI groups combined (from 0.42 at baseline to 0.15 at 12 months, −0.28) vs the CAU group (from 0.39 at baseline to 0.36 at 12 months, −0.03), as demonstrated in Fig. 3.

Consequently, the improvement in metabolic status over time resulted in a statistically significant positive effect on hyperandrogenism (estimate −0.812 s.e. 0.305, *P*  = 0.008). This effect could mainly be attributed to changes in biochemical hyperandrogenism (estimate −0.821 s.e. 0.259, *P*  = 0.002), whereas changes in clinical hyperandrogenism were non-significant (estimate −0.021 s.e. 0.262, *P*  = 0.936). And, although improvement of metabolic status showed a positively decreasing effect on ovulatory dysfunction, this was non-significant (estimate −0.442 s.e. 0.372, *P*  = 0.237). Additionally, there was no significant effect on PCOM (estimate 0.395 s.e. 0.540, *P*  = 0.466).

### *Post hoc* analysis; mediation

**Figure 4:**
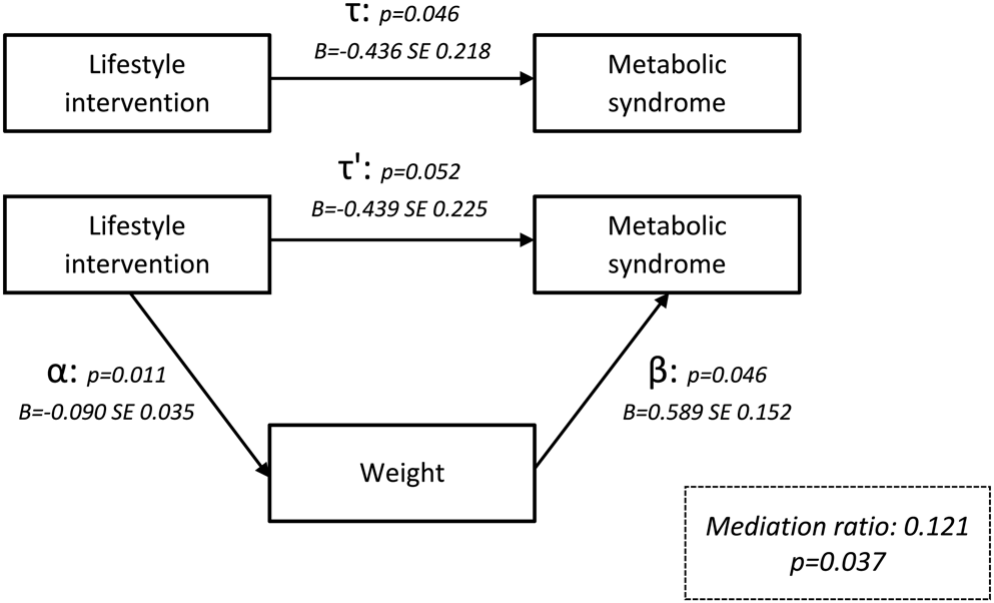
Mediation analysis demonstrating the effect of weight on the metabolic syndrome.

Weight, androgens (testosterone, androstenedione, dehydroepiandrosterone, dehydroepiandrosterone sulphate, and free androgen index), SHBG, insulin, and HOMA-IR were checked as mediating variables for the effect of the LSI on the prevalence of MetS. Weight resulted in mediation (mediation ratio 0.121, *P*  = 0.037) with pathways further specified in Fig. 4. This indicates that weight mediated the pathway between the LSI and MetS by 12.1%.

### *Post hoc* analysis; estimates of weight loss and weight gain *per se*

For the *post hoc* analyses on changes in weight *per se* on MetS and metabolic parameters, we pooled the effects of all three groups to evaluate all participants whose body weight was changed. The prevalence of MetS decreased by −13.2% when 5% weight loss was achieved and by −23.8% when participants achieved a 10% weight loss (estimate 0.114 s.e. 0.028, *P* < 0.001). The prevalence of MetS increased by +14.1% when participants gained 5% in weight. The separate NCEP diagnostic criteria for elevated WC, BP, and TGs also demonstrated a statistically significant interaction, as well as the prevalence of insulin resistance with results further specified in Table 4. Furthermore, changes in the percentage of body weight also had a statistically significant effect on the cMetS risk z-score (estimate 0.043 s.e. 0.005, *P* < 0.001). The same pattern was observed for all other continuous metabolic characteristics, especially if more weight loss was achieved. Weight gain worsened these metabolic parameters, see Table 4.

**Table 4:** Changes in metabolic syndrome and metabolic parameters after changes in body weight for all groups combined (SMS+, SMS−, and CAU). Differences were tested with multilevel logistic regression analyses for categorical variables and with multilevel linear regression analyses for continuous variables, combined with a bootstrap procedure in case of a non-normal distribution.

	Changes in body weight (all groups combined)				
	5% weight gain	5% weight loss	10% weight loss	Estimate (s.e.)	*P* value
Metabolic syndrome (%)	14.1	−13.2	−23.8	0.114 (0.028)	**<0.001**
Waist ≥ 88 cm (%)	5.5	−9.2	−22.9	0.139 (0.033)	**<0.001**
Glucose ≥ 6.1 mmol/L (%)	2.7	−1.7	−2.7	0.105 (0.061)	0.090
BP ≥ 129/84 mmHg (%)	6.9	−6.1	−11.4	0.061 (0.025)	**0.017**
HDL < 1.3 mmol/L (%)	1.4	−1.9	−4.4	0.066 (0.044)	0.133
TG ≥ 1.7 mmol/L (%)	5.5	−4.9	−9.0	0.054 (0.027)	**0.047**
Insulin resistance (%)	9.2	−11.5	−24.2	0.103 (0.025)	**<0.001**
Metabolic parameters					
cMetS z-score	0.22	−0.22	−0.43	0.043 (0.005)	**<0.001**
HOMA-IR	0.42	−0.42	−0.83	0.083 (0.017)	**<0.001**
SBP (mmHg)	2	−2	−4	0.373 (0.101)	**0.001**
DBP (mmHg)	2	−2	−3	0.333 (0.076)	**<0.001**
Waist (cm)	4.1	−4.1	−8.3	0.827 (0.080)	**<0.001**
Glucose (mmol/L)	0.1	−0.1	−0.2	0.015 (0.004)	**0.001**
Insulin (pmol/L)	11	−11	−22	2.187 (0.484)	**<0.001**
Cholesterol (mmol/L)	0.1	−0.1	−0.2	0.022 (0.006)	**0.001**
HDL (mmol/L)	−0.02	0.02	0.05	−0.005 (0.002)	**0.006**
LDL (mmol/L)	0.08	−0.08	−0.17	0.017 (0.005)	**0.005**
TG (mmol/L)	0.12	−0.12	−0.23	0.023 (0.005)	<0.001

BP, blood pressure; CAU, care as usual; cMetS z-score, continuous metabolic syndrome z-score; DBP, diastolic blood pressure; HDL, high-density lipoprotein; HOMA-IR, homeostatic model assessment for insulin resistance; LDL, low-density lipoprotein; SBP, systolic blood pressure; SMS−, lifestyle intervention without SMS support; SMS+, lifestyle intervention with SMS support; TG, triglyceride. Statistically significant values (*P<*0.05) are presented in bold.

## Discussion

This study analysed secondary outcome measures from a three-component long-term randomized controlled LSI in overweight and obese women with PCOS. The prevalence and severity of MetS decreased significantly in the group receiving the intervention, whereas the incidence of MetS increased in the group that received CAU. This effect was significantly mediated by weight, suggesting that the changes in MetS were related to the changes in weight through the LSI. Moreover, improvement in metabolic status had a positive effect on (biochemical) hyperandrogenism. BP, WC, cholesterol, and LDL decreased, and HDL increased within all groups. However, positive changes were more evident within the LSI groups. Furthermore, weight loss, in general, resulted in positive effects on the prevalence and severity of MetS as well as on all metabolic characteristics separately.

Our study demonstrated favourable changes concerning metabolic health. These changes were very much determined by the extent of advice and guidance provided in the lifestyle programme as well as by the amount of weight loss achieved in that programme. Similarly, positive effects of LSIs on total cholesterol, LDL, and fasting insulin levels have been demonstrated before (21). These studies also demonstrated the important positive effect of exercise on insulin resistance (33). Not many participants in our RTC achieved a 5–10% weight loss (32), which may possibly explain the small between-group effects with regard to changes in metabolic parameters. The large number of dropouts is a common problem in LSI programmes, and this was unfortunately not different in our study. However, and although in only a small group of patients, we do provide robust evidence that supports the recommendation for achieving a minimum of 5–10% sustainable weight loss in future LSIs, as described in the current PCOS guideline (1).

Different ways have been found to assess the severity status of MetS and possible future metabolic diseases. For example, the within-group decreases in our study, especially those in BP and WC, may prevent severe future metabolic complications such as cardiovascular disease and/or events. This can be concluded because Guize and colleagues investigated combinations of the different metabolic components and observed a statistically significant high risk of all-cause mortality for MetS diagnoses in which elevated WC and elevated BP play key roles (34). This finding indicates the need for the elimination of these features. Another measure to evaluate the severity status of MetS is the cMetS z-score. Our study demonstrated a significant decrease in the severity score in the LSI groups, which further emphasizes the positive effect of LSIs on metabolic health. The cMetS severity score has also proven to possess predictive ability for future coronary heart disease (28) and type 2 diabetes mellitus (27) in the general population. We believe that it could be very helpful in predicting similar risks in women with PCOS as well.

However, even though women with PCOS exhibit metabolic derangements at a young age, which is believed to worsen with ageing (35) and excess weight (9), controversy still remains about the long-term risk of cardiovascular disease in women with PCOS. Despite this controversy, we cannot ignore the metabolic derangement in reproductive-aged women with PCOS, since its impact on their cardiovascular health is likely similar to its impact on women without PCOS. Hence, lifestyle changes are needed to improve metabolic health in these women. Moreover, women with PCOS present more often with pregnancy-related complications such as gestational diabetes, pregnancy-induced hypertension, pre-eclampsia, and preterm birth (36). Such complications might also be prevented by optimizing the metabolic pre-pregnancy condition with the use of an LSI.

A major strength of our study is that this is the largest three-component LSI study cohort of women with PCOS to date. Another strength of this study is that we used the cMetS z-score for the first time in a cohort of reproductive-aged women with PCOS. Traditional MetS criteria compose a binary classification; MetS is either present or it is not. An advantage of the cMetS z-score is the identification of individuals who are at high risk for developing MetS. These individuals may have MetS diagnostic measurements with elevations that are just below cut-off values. The traditional MetS criteria would have labelled these individuals as low-risk (25). Moreover, this individual severity score makes it possible to follow changes over time and to assess the effects of certain LSIs, as demonstrated by the current RCT.

Dropout during LSIs is unfortunately a common phenomenon, and our RCT also suffered from considerable discontinuation rates, which is a limitation. Participant-related factors which predict dropout at baseline have not yet been identified. Especially, study duration seems to be a factor with a particularly negative effect on adherence (37). Because of the length of our study, the intensity of the programme and the fact that spontaneous pregnancies were a reason to discontinue study participation, we expected to have high discontinuation rates and anticipated this with the sample size calculation (22). Also, we selected a statistical method, for example multilevel regression modelling, specifically designed to deal with such missing values (31). Despite these missing values, data from this RCT provide valuable information on the effect of a three-component LSI. Also, women who completed the programme will have a substantial advantage in managing a healthy lifestyle. Nonetheless, future studies should focus on strategies to increase adherence rates.

## Conclusion

This three-component lifestyle RCT demonstrated statistically significant and clinically relevant improvements in metabolic health. Notwithstanding the high dropout rate, three-component LSIs aiming at a 5–10% weight loss should be recommended for all women with PCOS in order to improve metabolic health during their reproductive lifespan.

## Declaration of interest

A D L, G J, A B, and J B have nothing to declare. J L reports grants from Ansh Labs, Webster, Tx, USA, grants from Ferring, Hoofddorp, NL, grants from Dutch Heart Association, Utrecht, NL, grants from Zon MW, Amsterdam, NL, grants from Astellas, Tokyo, Japan, grants from Roche Diagnostics, Basel, Switzerland, personal fees from Ferring, Hoofddorp, NL, personal fees from Titus Healthcare, Hoofddorp, NL and is an unpaid board member and president-elect of the AE-PCOS Society, outside the submitted work.

## Funding

This work did not receive any specific grant from any funding agency in the public, commercial or not-for-profit sector.
